# Properties of High-Density Fiberboard Bonded with Urea–Formaldehyde Resin and Ammonium Lignosulfonate as a Bio-Based Additive

**DOI:** 10.3390/polym13162775

**Published:** 2021-08-18

**Authors:** Petar Antov, Viktor Savov, Neno Trichkov, Ľuboš Krišťák, Roman Réh, Antonios N. Papadopoulos, Hamid R. Taghiyari, Antonio Pizzi, Daniela Kunecová, Marina Pachikova

**Affiliations:** 1 Faculty of Forest Industry, University of Forestry, 1797 Sofia, Bulgaria; victor_savov@ltu.bg (V.S.); ntrichkov@ltu.bg (N.T.); 2 Faculty of Wood Sciences and Technology, Technical University in Zvolen, 96001 Zvolen, Slovakia; reh@tuzvo.sk; 3 Laboratory of Wood Chemistry and Technology, Department of Forestry and Natural Environment, International Hellenic University, GR-661 00 Drama, Greece; antpap@for.ihu.gr; 4 Wood Science and Technology Department, Faculty of Materials Engineering & New Technologies, Shahid Rajaee Teacher Training University, Tehran 16788-15811, Iran; htaghiyari@sru.ac.ir; 5 LERMAB-ENSTIB, University of Lorraine, 27 Rue Philippe Seguin, 88000 Epinal, France; antonio.pizzi@univ-lorraine.fr; 6 Faculty of Engineering, Slovak University of Agriculture in Nitra, 94976 Nitra, Slovakia; daniela.kunecova@uniag.sk; 7 Kronospan Bulgaria EOOD, 5000 Veliko Tarnovo, Bulgaria; m.pachikova@kronospan.bg

**Keywords:** wood-based panels, high-density fiberboards, bio-based adhesives, urea–formaldehyde resin, ammonium lignosulfonate, formaldehyde emission

## Abstract

The potential of ammonium lignosulfonate (ALS) as an eco-friendly additive to urea–formaldehyde (UF) resin for manufacturing high-density fiberboard (HDF) panels with acceptable properties and low free formaldehyde emission was investigated in this work. The HDF panels were manufactured in the laboratory with very low UF resin content (4%) and ALS addition levels varying from 4% to 8% based on the mass of the dry wood fibers. The press factor applied was 15 s·mm^−1^. The physical properties (water absorption and thickness swelling), mechanical properties (bending strength, modulus of elasticity, and internal bond strength), and free formaldehyde emission were evaluated in accordance with the European standards. In general, the developed HDF panels exhibited acceptable physical and mechanical properties, fulfilling the standard requirements for HDF panels for use in load-bearing applications. Markedly, the laboratory-produced panels had low free formaldehyde emission ranging from 2.0 to 1.4 mg/100 g, thus fulfilling the requirements of the E0 and super E0 emission grades and confirming the positive effect of ALS as a formaldehyde scavenger. The thermal analyses performed, i.e., differential scanning calorimetry (DSC), thermal gravimetric analysis (TGA), and derivative thermogravimetry (DTG), also confirmed the main findings of the research. It was concluded that ALS as a bio-based, formaldehyde-free adhesive can be efficiently utilized as an eco-friendly additive to UF adhesive formulations for manufacturing wood-based panels under industrial conditions.

## 1. Introduction

Due to recent technological and material developments, the wood-based panel industry has become one of the fastest growing industries worldwide [1,2] with an estimated annual production of approximately 357 million m^3^ in 2019, which represents a 100% increase compared to 2000 [3]. The production of medium-density fiberboard (MDF) and high-density fiberboard (HDF) panels, with a total global production of more than 105 million m^3^ in 2019, is the second largest worldwide, preceded only by plywood manufacturing [3]. The transition to circular, low-carbon bioeconomy, growing environmental concerns, and strict legislation related to emission of harmful volatile organic compounds (VOCs), such as free formaldehyde from wood-based panels, have posed new requirements related to the development of environmentally friendly wood-based panels [4,5,6,7,8,9], optimization of lignocellulosic resources [10,11,12,13], and use of alternative raw materials [14,15,16,17,18,19].

Conventional thermosetting formaldehyde-based wood adhesives, which represent approximately 95% of the total adhesives used in the wood-based panel industry, are commonly made from fossil-derived constituents, such as urea, phenol, melamine, etc. [20,21,22,23]. Due to their numerous advantages, such as short press times, low press temperatures, high reactivity, water solubility, good adhesion performance, and low price [23,24,25,26], urea–formaldehyde (UF) resins have become the most important type of resins used in the production of wood-based panels with an estimated global annual consumption of approximately 11 million tons of resin solids [25,26,27]. However, the main disadvantage of these thermosetting aminoplastic resins, besides the deteriorated water resistance [28], is the emission of hazardous VOCs, including free formaldehyde release from the finished wood-based panels [29,30], which is related to a number of serious environmental problems and adverse effects on human health, such as skin and eye irritation, respiratory problems, and cancer [31,32,33]. Harmful formaldehyde emissions can be reduced by adding various inorganic, organic, and mineral compounds as formaldehyde scavengers to conventional wood adhesives, such as phosphates, salts, urea, nanoparticles, bark, tannins, etc. [34,35,36,37,38,39,40,41,42,43], by surface treatment of finished wood-based panels [34] or by using eco-friendly, formaldehyde-free adhesive formulations [44,45,46,47,48,49,50,51,52,53,54].

Successful attempts have been made to develop eco-friendly wood panels bonded with sustainable “green” adhesives based on modified tannins [55,56,57], soy [58,59,60], starch [61,62], and lignin [63,64,65,66,67,68,69]. A common disadvantage of all bio-based binders is the need to apply extended press times and the deteriorated dimensional stability of the ready panels [70,71].

Lignin is the largest renewable aromatic polymer in nature and the second most abundant organic material on the planet, preceded only by cellulose [72,73]. Each year, about 50–70 million tons of lignin are obtained as waste or by-product of the pulp and paper industry alone, of which less than 2% is used for manufacturing value-added products [72], while the rest is primarily used for production of energy [72,73,74,75]. Considering the increased production of bioethanol, the total amount of lignin is expected to reach 225 million tons by 2030 [72,73,74,75,76]. Therefore, it is important to identify and develop new approaches and industrial applications of this underutilized raw material. The valorization of lignin as a renewable feedstock for the production of valued-added, bio-based chemicals, including wood adhesives, has gained significant industrial and scientific interest due to its annual renewability and phenolic structure, allowing partial replacement of phenol in phenol–formaldehyde (PF) resins [77,78,79,80,81]. In addition, lignin contains methoxyl, carbonyl, and aliphatic hydroxyl groups that are favorable for modifications, e.g., methylolation and phenolation, aimed at increasing its chemical reactivity to formaldehyde [82].

The extraction of lignin from lignocellulosic biomass can be performed by several technological methods (enzymatic, chemical, and mechanical) to produce different technical lignins. The Kraft pulping process [83], which produces alkali lignin, and sulfite pulping, which produces lignosulfonates [74], are considered among the most effective methods for separation of lignin from wood. Lignosulfonates (LS), obtained using sulfurous acid and sulfite or bisulfite salts containing ammonium, magnesium, calcium, or sodium at different pH levels [84], represent a major share of the total technical lignins produced worldwide with a reported annual production of 1.8 million tons [74]. The water solubility of LS due to the higher degree of sulfonation compared with Kraft lignin, their relatively high molecular weight, and the availability of a large number of functional groups are the main factors affecting the application of LS in wood adhesives. Ammonium lignosulfonates (ALS) are one of the most promising lignin-based products for wood adhesive applications due to increased presence of hydroxyl groups [85,86] and their solubility in organic solvents [86].

The aim of this research study was to investigate the potential of decreasing the UF resin content by incorporating ALS to the adhesive formulation in order to produce low-toxic, environmentally friendly HDF panels from industrial hardwood fibers with physical and mechanical properties complying with the European standard requirements under industrial conditions.

## 2. Materials and Methods

Industrially produced wood fibers, obtained in factory conditions by the Asplund method using the Defibrator L 46 (Valmet, Stockholm, Sweden) equipment and composed of two hardwood species, namely European beech (*Fagus sylvatica* L.) and Turkey oak (*Quercus cerris* L.) at the ratio 2:1, oven dried to 6.1% moisture content, were provided by the factory Welde Bulgaria AD (Troyan, Bulgaria). Fibers were produced by thermomechanical defibration of wood chips by applying steam treatment at a pressure of 0.8 MPa and temperature of 170 °C. The fibers had the following characteristics (factory data): lengths ranging from 1115 to 1260 µm, pulp freeness of 11° SR as determined by the Schopper–Riegler test, and a bulk density of 27 kg·m^−3^.

The adhesive formulation comprised commercial UF resin (64% dry solids content, 1.16 molar ratio), supplied by the factory Kastamonu Bulgaria AD (Gorno Sahrane, Bulgaria), and a novel ALS additive named D-947L, a product of Borregaard (Sarpsborg, Norway), which had the following properties: total solids content: 48.6%, ammonium content: 4.1%, sodium content: 0.1%, total sulfur content: 6.8%, sugars: 20%, viscosity (cps): 400 at 25 °C, pH: 4.5%, specific gravity: 1.220 g·cm^−3^, and boiling point: 104 °C.

The dynamic viscosity (mPa.s) of the adhesive formulation was measured using the DV2T-LV rotary viscometer (Brookfield, Middleboro, MA, USA) equipped with a coaxial cylinder precision sample adapter and standard spindle ULA following the standard test method [87]. The viscosity of the UF resin and UF + ALS adhesive mixtures was measured in a torque rate between 30% and 40% at the standard room temperature of 22 °C.

In the laboratory, the HDF panels with dimensions of 400 mm × 400 mm × 6 mm and target density of 910 kg·m^−3^ were fabricated. In our previous study [66], HDF panels bonded with UF resin and ALS and manufactured in the laboratory at a press factor of 30 s·mm^−1^ had satisfactory physical and mechanical properties and a markedly low formaldehyde content ranging from 0.7 to 1.0 mg/100 g, in accordance with the standard Perforator method [88]. Thus, in this study, in order to attain the common industrial practice, we tried to further optimize the press factor by applying adhesive formulation composed of UF resin at 4% and three different additions levels (4%, 6%, and 8%) of ALS based on the dry weight of fibers. The UF resin was used at 50% concentration.

Urea (CH_4_N_2_O) at 3% based on dry resin and applied at 30% concentration was used as a formaldehyde scavenger. Ammonium sulfate ((NH4)_2_SO_4_) at 2% based on dry ALS and at 1.5% based on dry UF resin was used as a hardener at 30% concentration.

The manufacturing parameters of the laboratory-produced HDF panels are given in Table 1.

Two control panels (REF 4 and REF 6) bonded with 4% and 6% UF resin based on the dry fibers and without ALS were fabricated. The first control panel (REF 4) was used to evaluate the effect of adding ALS on the panel properties. The second control panel (REF 6) was manufactured with a resin addition level of 6%, which is typical for commercially produced HDF panels [22,89].

Wood fibers were mixed with the adhesive in a high-speed laboratory blender with needle-shaped paddles (prototype, University of Forestry, BG) at 850 min^−1^. The UF resin or the adhesive mixture of UF resin + ALS was sprayed in the laboratory blender through a 1.5 mm nozzle, followed by injecting a paraffin emulsion (solid content: 60 ± 2%, melting point: 62 °C, oil content: 5–7%, specific gravity: 0.95 g·cm^−3^) as a hydrophobing agent. The hot-pressing process was performed in a single opening hydraulic press (PMC ST 100, Italy). The press temperature used was 220 °C. The press factor applied was 15 s·mm^−1^, which is very close to the industrial practice. A four-stage pressing regime with the following pressure values was used: (i) 4.5 MPa for 15 s, (ii) 1.2 MPa for 15 s, (iii) 0.6 MPa for 30 s, and (iv) 1.8 MPa for 30 s. Following the hot pressing, the manufactured panels were conditioned for 7 days at a temperature of 20 ± 2 °C and 65% relative humidity. The pressing regime was selected in accordance with the common industrial practice for production of HDF panels [90].

**Figure 1:**
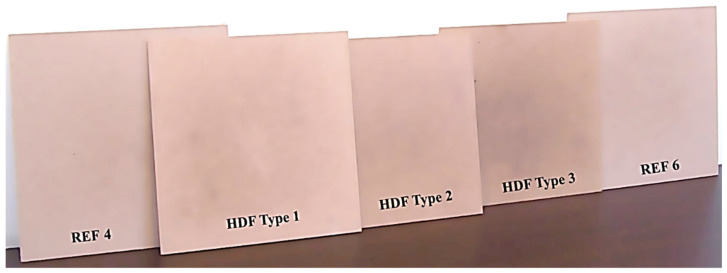
HDF panels from industrial hardwood fibers bonded with UF resin and ALS (910 kg·m^−3^ target density, 6 mm thickness, and three addition levels (4%, 6%, and 8%) of ALS) and control HDF panels (REF 4 and REF 6) bonded with UF resin.

The physical and mechanical properties of the laboratory-produced HDF panels (Figure 1) were determined according to the standards EN 310, EN 317, EN 322, and EN 323 [91,92,93,94]. The mass of the test specimen was measured using a precision laboratory balance Kern (Kern & Sohn GmbH, Balingen, Germany) with an accuracy of 0.01 g. The dimensions of the test samples were measured using digital calipers with an accuracy of 0.01 mm. Water absorption and thickness swelling tests were carried out by the weight method after 24 h of immersion in water [92]. The mechanical property tests were performed on universal testing machine Zwick/Roell Z010 (Zwick/Roell GmbH, Ulm, Germany). For each property, eight HDF test samples were used for testing.

The formaldehyde content of the laboratory-fabricated panels was measured in the laboratory of Kronospan Bulgaria EOOD (Veliko Tarnovo, Bulgaria) on four test samples in accordance with the standard EN ISO 12460-5 [88].

For thermal analyses, DSC (differential scanning calorimetry), TGA (thermal gravimetric analysis), and DTG (derivate thermogravimetry) were used. The samples for thermal analyses were weighed by scales KERN ABT 220-5DM version 1.2 03/2013 (Kern & Sohn GmbH, Balingen, Germany).

The curing process was monitored using DSC 1 (Mettler-Toledo GmbH, Greifensee, Switzerland) equipment. Nitrogen (purity 99%) was used as a carrier gas, and the gas flow rate was 20 mL·min^−1^. During analysis, the DSC instrument lid was not pierced and was fixed on crucible. This method provides the ability to monitor endothermic and exothermic processes corresponding to the peaks and changes the shape of DSC curves (onset temperature and temperature of peak). Samples were heated from 25 to 210 °C at a heating rate of 10 °C/min under a nitrogen flow rate of 50 mL/min. Approximately 25 mg of oven-dried samples was used to carry out each analysis. Curve analysis was performed using the STAR^® 9.3^ (Mettler-Toledo GmbH, Greifensee, Switzerland) thermal analysis evaluation software.

TGA was used to monitor changes in the weight of the adhesive formulation samples as a function of temperature. This method provides information on thermal oxidative degradation rates and temperatures of polymeric materials. TGA was used together with DTG to identify and quantitatively analyze the chemical composition of substances. The device Mettler Toledo TGA/DSC 2 (Mettler-Toledo GmbH, Greifensee, Switzerland) was used to analyze the thermal stability of adhesive samples. TGA and DTG measurements were carried out in an alumina crucible with lids, diameter of 6 mm, and length of 4.5 mm. Around 20 mg of each adhesive formulation was placed in a 70 µL crucible and heated from room temperature to 200 °C at the heating rate of 10 °C/min under a flowing nitrogen atmosphere. Gas flow was 20 mL·min^−1^. The isothermal part occurred after reaching 200 °C in 5 min, then increased to 210 °C at the rate 15 °C/min.

Variation and statistical analyses of the results were performed using the specialized QstatLab 6.0 software. Hierarchical cluster analysis was carried out by SPSS/18 software (IBM, USA, 2009) to categorize the treatments and to find out similarities based on all properties measured in this study. Contour and surface plots were designed using Minitab software, version 16.2.2 (2010; Minitab Inc., State College, PA, USA).

## 3. Results and Discussion

### 3.1. Adhesive Mixture Viscosity

The dynamic viscosity (mPa.s) of the UF resin and adhesive mixtures used (UF resin + ALS) has a strong effect on its application to wood fibers. Different average dynamic viscosity values were determined with different addition levels (4%, 6%, and 8%) of ALS (Table 2).

The results showed that the addition of ALS to the UF resin resulted in increased dynamic viscosity values of the adhesive mixture. At all addition levels of ALS, the adhesive mixture was homogenous and easy to apply to wood fibers.

### 3.2. Physical and Mechanical Properties

The results obtained for the physical and mechanical properties of the laboratory-fabricated HDF panels bonded with adhesive formulation comprising UF resin and ALS (D-947L) are presented below. The density of the HDF panels, presented in Table 3, varied from 910 to 917 kg·m^−3^, i.e., close to the targeted value of 910 kg·m^−3^.

The difference between the panel with the highest density value (HDF Type 3) and the panel with the lowest determined density (HDF Type 2) was below 0.8%. The calculated *p*-value of the *t*-test performed between the respective density values was 0.71, i.e., greater than 0.05. Thus, it can be concluded that the difference between the densities was not statistically significant.

Water absorption (WA) and thickness swelling (TS) are critical physical properties of wood-based panels and are strongly correlated with the dimensional stability of laboratory-fabricated HDF panels [20,95,96,97]. Both properties were evaluated after 24 h of soaking in water.

**Figure 2:**
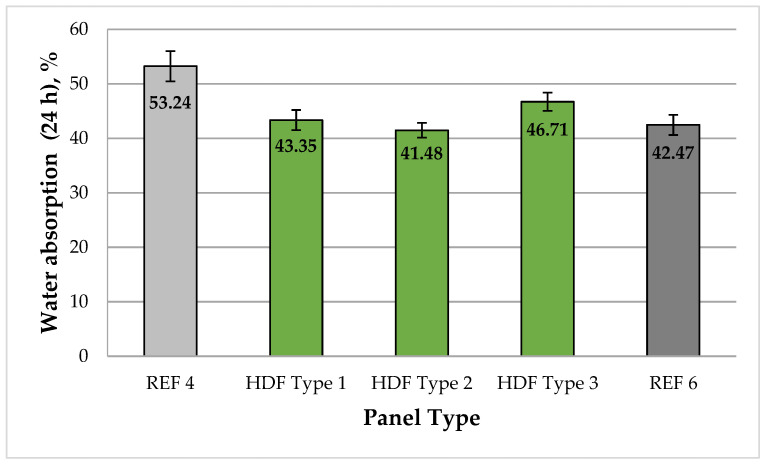
Water absorption (24 h) of the HDF panels produced. REF 4: 4% UF resin; HDF Type 1: 4% UF resin and 4% ALS; HDF Type 2: 4% UF resin and 6% ALS; HDF Type 3: 4% UF resin and 8% ALS; and REF 6: 6% UF resin. (Error bar represents the standard deviation.).

A graphical representation of the WA (24 h) of the laboratory-fabricated HDF panels is shown in Figure 2.

WA values of the laboratory-produced HDF panels varied from 53.2% to 41.5%. The highest WA values were obtained for the control panel fabricated with UF resin (REF 4), and the lowest values were obtained for the panel bonded with 4% UF resin and 6% ALS (HDF Type 2). The HDF panels produced with 4% ALS and 6% ALS had 1.23 times and 1.28 times lower WA values than the control panel (REF 4), respectively. In addition, these laboratory panels had WA values comparable with the control panel fabricated with 6% UF resin (REF 6). The HDF panel fabricated with 8% ALS was characterized by 1.13 times higher WA values compared with the panel bonded with 6% ALS addition. This might be attributed to the greater moisture content of the wood fibers caused by the increased ALS content, which led to reduced bonds between the lignosulfonate and hardwood fibers for the conditions used in the experiment (press factor of 15 s·mm^−1^). The WA values obtained were significantly better in comparison with previous studies on the use of lignosulfonates as bio-based, formaldehyde-free wood adhesives [66,67,68,69,70,71,97,98,99].

WA is not a standardized physical property of wood-base panels. According to literature data [22,99] common HDF panels have WA values typically ranging from 30% to 45%. Thus, the HDF panels produced in the laboratory from industrial hardwood fibers and bonded with an adhesive formulation comprising UF resin and ALS addition of 4% and 6% demonstrated WA values comparable with industrial HDF panels bonded with conventional synthetic adhesives [100,101].

**Figure 3:**
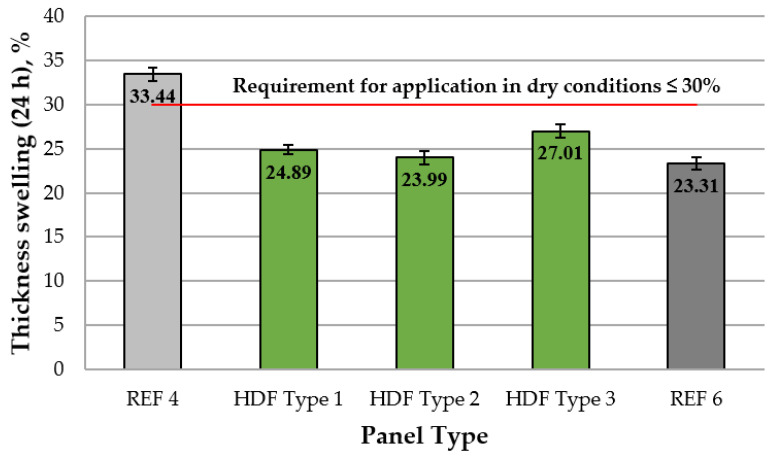
Thickness swelling (24 h) of HDF panels produced. REF 4: 4% UF resin; HDF Type 1: 4% UF resin and 4% ALS; HDF Type 2: 4% UF resin and 6% ALS; HDF Type 3: 4% UF resin and 8% ALS; and REF 6: 6% UF resin. (Error bar represents the standard deviation, and the red line represents the EN 622-5 standard requirement for load-bearing panels for use in dry conditions [102]).

A graphical representation of the TS (24 h) of the laboratory-fabricated HDF panels is shown in Figure 3.

As seen in Figure 3, the TS values of laboratory-fabricated HDF panels ranged from 33.4% to 23.3%. The addition of 4% ALS resulted in significantly improved (lower) TS values by 1.34 times compared to the control panel REF 4. Increasing the ALS content from 4% to 6% led to slightly better TS values that was comparable with the respective values of the control panel produced with 6% UF resin (REF 6). The HDF panel bonded with 8% ALS exhibited deteriorated (increased) TS values. This confirmed that increasing the ALS content over 6% at the press factor of 15 s·mm^−1^ resulted in deteriorated water-related properties of the panels. The high TS values may also be explained by the highly hygroscopic nature of fiberboard panels [103,104].

The TS values of the laboratory-made HDF panels were significantly better compared to the results obtained in previous studies on the application of lignosulfonates as adhesives in wood composites [66,78,97,98]. The HDF panels produced in this work with 4% and 6% ALS addition levels showed TS values comparable with the results reported in previous studies using UF resin with a similar molar ratio (1.17) [100] or UF resin modified with melamine [101]. Markedly, all laboratory-produced HDF panels had considerably better TS values than the standard requirement for load-bearing panels for use in dry conditions, i.e., 30% [102].

In terms of mechanical properties, the modulus of elasticity (MOE), bending strength (MOR), and internal bond (IB) strength of the laboratory-fabricated HDF panels were determined.

**Figure 4:**
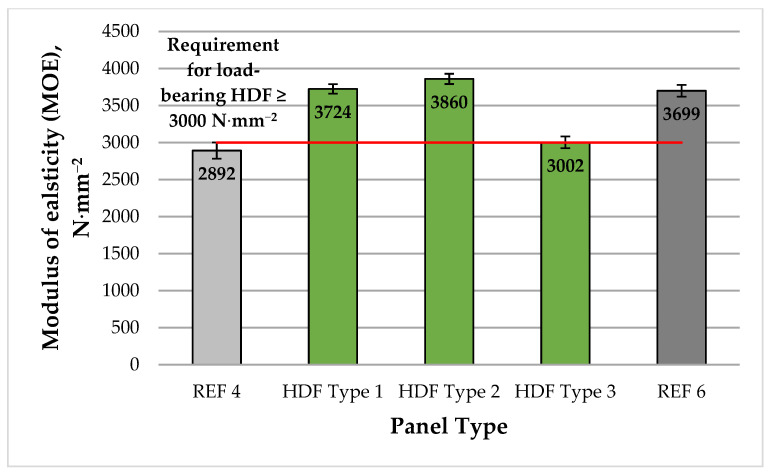
Modulus of elasticity (MOE) of HDF panels produced. REF 4: 4% UF resin; HDF Type 1: 4% UF resin and 4% ALS; HDF Type 2: 4% UF resin and 6% ALS; HDF Type 3: 4% UF resin and 8% ALS; and REF 6: 6% UF resin. (Error bar represents the standard deviation, and the red line represents the EN 622-5 standard requirement for panels for use in load-bearing applications [102]).

A graphical representation of the mean MOE values of the different HDF panels produced in this work is shown in Figure 4.

The HDF panels bonded with UF resin and ALS as an eco-friendly additive exhibited MOE values ranging from 2892 to 3860 N·mm^−2^. The estimated MOE values of the laboratory panels fabricated with 4% and 6% ALS addition levels surpassed the most stringent European standard requirement for panels used in load-bearing applications (≥3000 N·mm^−2^) [102] by 24.1% and 28.7%, respectively, and had greater MOE values than the control panel produced with 6% UF resin only (REF 6). This may be attributed to the increased elasticity of the panels bonded with bio-based adhesives and especially with lignin-based binders [105]. The addition of 4% ALS to the UF resin resulted in improved MOE values compared with the control HDF panel fabricated with 4% UF resin as a binder (REF 4). Increasing the ALS content from 4% to 6% resulted in a slightly improved MOE values by 3.7%. Further increase of ALS addition to 8% resulted in deteriorated (lower) MOE values by 22% compared with the panel bonded with 6% ALS, i.e., the applied press factor of 15 s·mm^−1^ was not sufficient for the creation of adequate adhesive bonds between the ALS and wood fibers. The determined MOE values of the laboratory panels were comparable or greater than the results reported in previous studies on the application of lignosulfonates as wood adhesives [78,97,98], achieved at almost 9 times shorter press factor and without further processing of the panels. Comparable MOE values were reported by the authors of [66] in their research on the development of environmentally friendly HDF panels bonded with low UF resin content (3%) and ALS addition levels ranging from 6% to 10% based on dry fibers but at a press factor twice as long as that applied here. In the same work, the authors reported higher MOE values of the panels achieved at 10% ALS content.

**Figure 5:**
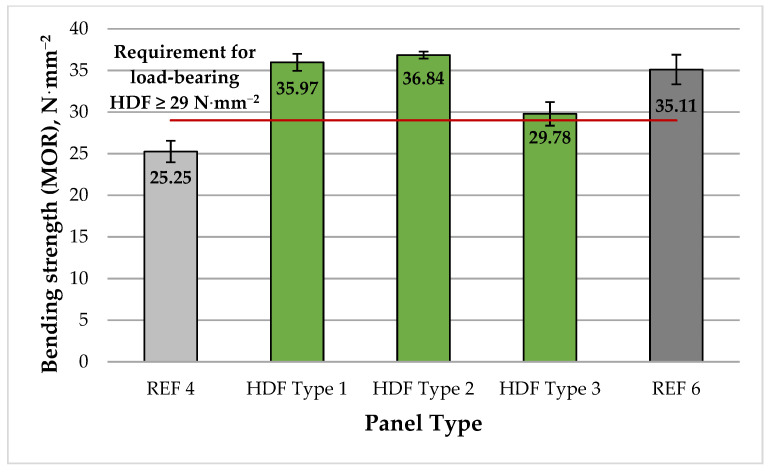
Bending strength (MOR) of HDF panels produced. REF 4: 4% UF resin; HDF Type 1: 4% UF resin and 4% ALS; HDF Type 2: 4% UF resin and 6% ALS; HDF Type 3: 4% UF resin and 8% ALS; and REF 6: 6% UF resin. (Error bar represents the standard deviation, and the red line represents the EN 622-5 standard requirement for panels for use in load-bearing applications [102].).

A graphical representation of the MOR values of the laboratory-produced HDF panels is presented in Figure 5.

The HDF panels exhibited MOR values ranging from 25.25 to 36.84 N·mm^−2^. According to [106], MOE and MOR values of HDF panels are strongly correlated with the fiber dimensions. The authors pointed to the wood species and the digester technical parameters, i.e., press temperature, pressure applied, and time, as the most important factors affecting the physical and mechanical properties of HDF panels. In addition, MOE and MOR values of fiberboards are positively affected by increased panel density [107,108]. The maximum MOR value achieved in this work, i.e., 36.84 N·mm^−2^, was determined at 4% UF resin content and 6% ALS addition (HDF Type 2 panel). The addition of 4% ALS to the UF resin resulted in significant improvement of the MOR values by almost 43% compared to the control panel fabricated with 4% UF resin (REF 4). Increasing the ALS content from 4% to 6% resulted in a slightly improved MOR values by 2.4%. The laboratory panels fabricated with 4% and 6% ALS content exceeded the EN 622-5 standard requirements for HDF panels for use in load-bearing applications (≥29 N·mm^−2^) [102] by 24% and 27%, respectively, and had slightly greater MOR values than the control panel manufactured with 6% UF resin (REF 6).

**Figure 6:**
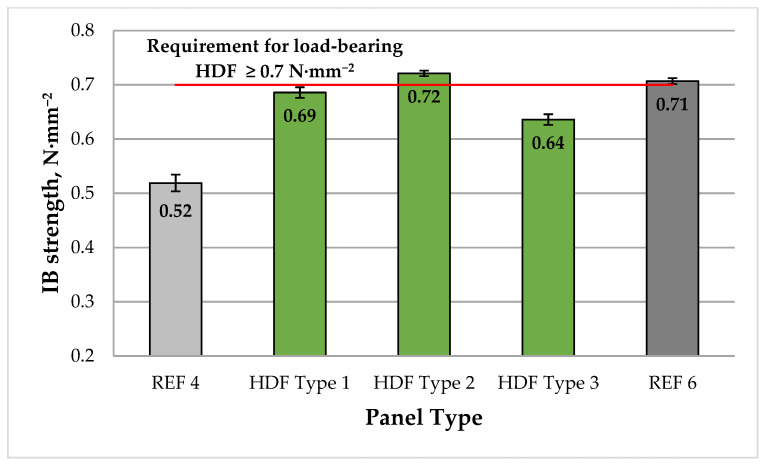
Internal bond (IB) of HDF panels produced. REF 4: 4% UF resin; HDF Type 1: 4% UF resin and 4% ALS; HDF Type 2: 4% UF resin and 6% ALS; HDF Type 3: 4% UF resin and 8% ALS; and REF 6: 6% UF resin. (Error bar represents the standard deviation, and the red line represents the EN 622-5 standard requirement for panels for use in load-bearing applications [102].).

A graphical representation of the mean IB strength of the laboratory-produced HDF panels is shown in Figure 6.

The IB values of HDF panels varied from 0.52 to 0.72 N·mm^−2^. The addition of 4% ALS to the UF resin resulted in significantly improved IB values by approximately 33% compared with the control panel bonded with 4% UF resin and without ALS (REF 4). Similar to the MOE and MOR results, increasing the ALS content from 4% to 6% resulted in a slightly improved IB values by 4.4%. The HDF panels produced with 4% and 6% ALS addition exhibited IB values rather close to the most stringent European standard requirement for HDF panels for use in load-bearing applications (≥0.7 N·mm^−2^) [102]. Only the laboratory panel manufactured with 6% ALS met this requirement. As reported in [109], the addition of ALS significantly reduces the interfacial tension and free energy, thus improving the mechanical properties of wood-based panels. The panel manufactured with 8% ALS exhibited deteriorated IB values, which confirmed that the press factor of 15 s·mm^−1^ was insufficient for this ALS addition level. In general, the IB values of HDF panels produced in this work were comparable or slightly higher than the values reported in [66], which might be attributed to the increased UF resin content from 3% to 4%. The results obtained are also comparable to similar studies using conventional synthetic resins [101]. According to previous research [110,111], the IB of fiberboards is mostly affected by the core density and not by the processing parameters.

**Figure 7:**
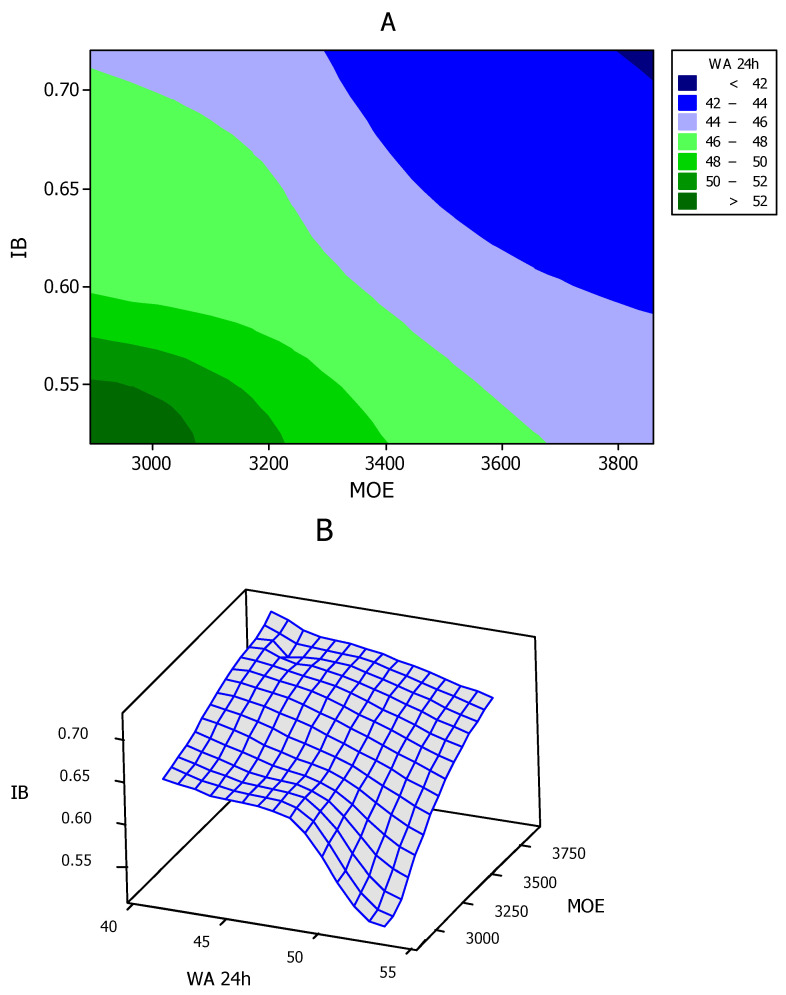
Contour (**A**) and surface (**B**) plots in the six different HDF panels produced. REF 4: 4% UF resin; HDF Type 1: 4% UF resin and 4% ALS; HDF Type 2: 4% UF resin and 6% ALS; HDF Type 3: 4% UF resin and 8% ALS; and REF 6: 6% UF resin. (IB = internal bond; MOE = modulus of elasticity; WA 24 h = water absorption after 24 h of immersion in water).

**Figure 8:**
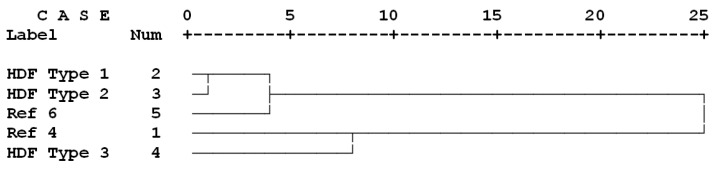
Cluster analysis based on all physical and mechanical properties as well formaldehyde emission between the six different HDF panels produced. REF 4: 4% UF resin; HDF Type 1: 4% UF resin and 4% ALS; HDF Type 2: 4% UF resin and 6% ALS; HDF Type 3: 4% UF resin and 8% ALS; and REF 6: 6% UF resin.

Contour and surface plots illustrated a smooth trend and high relationship between the physical and mechanical properties measured in the present research study (Figure 7A,B). The clear decreasing trend in WA values in the contour plot (Figure 7A) was associated with the increasing trend in both mechanical properties of IB and MOE, indicating that utilization of proper ALS content (up to 6%) would provide improved effects on all physical and mechanical properties. Moreover, cluster analysis demonstrated close clustering of Type 1 and 2 panels with panel REF 6 (Figure 8). This indicated that one of the main objectives of the present study was achieved, that is, addition of ALS improved the physical and mechanical properties of panels with only 4% UF resin to levels similar to those of panels with 6% UF resin (panel type REF 6). Cluster analysis also showed close clustering of Type 3 panels (with 8% ALS) with REF 4 panels. This clearly indicates that the improving effect of ALS is dependent on its content level, that is, in HDF panels with 4% UF resin, an increase in binding and properties can be expected in panels with up to 6% ALS content, while a further increase in ALS content would result in deterioration of the properties. Based on the results of the tests, it can be concluded that Type 2 HDF panels with 4% UF resin and 6% ALS can be recommended to achieve optimum physical and mechanical properties as well as reduce formaldehyde emission.

### 3.3. Free Formaldehyde Content

**Figure 9:**
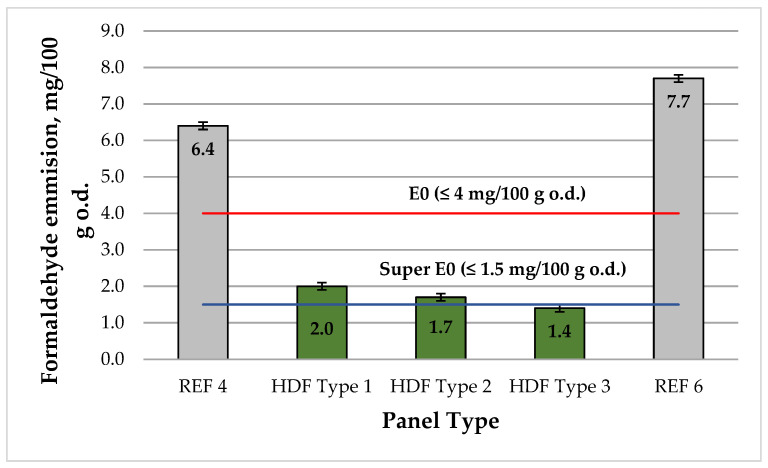
Free formaldehyde emission in the HDF panels produced: REF 4: 4% UF resin; HDF Type 1: 4% UF resin and 4% ALS; HDF Type 2: 4% UF resin and 6% ALS; HDF Type 3: 4% UF resin and 8% ALS; and REF 6: 6% UF resin. (Error bar represents the standard deviation, the red line represents the emission class E0 requirement, and the blue line represents the requirement of the emission class super E0.).

The results for the free formaldehyde content of the laboratory-fabricated HDF panels, determined in accordance with the standard EN ISO 12460-5 (called the Perforator method) [88], are presented in Figure 9.

All laboratory-produced HDF panels from industrial wood fibers bonded with UF resin and ALS (D-947L) exhibited remarkably low formaldehyde content, fulfilling the requirements of the E0 emission category, i.e., ≤4 mg/100 g oven dry (o.d.) [5,52]. The lowest formaldehyde content of 1.4 ± 0.1 mg/100 g was measured for the HDF panel bonded with 4% UF resin and 8% ALS addition level, fulfilling the requirements of the super E0 emission class (≤1.5 mg/100 g). This also confirmed that the ALS additive acted as a formaldehyde scavenger [42]. Due to the high amount of phenolic hydroxyl groups, ALS has very good reactivity towards formaldehyde [109,112,113]. According to the formaldehyde content results, the control HDF panels bonded with 4% and 6% UF resin only were classified under emission grade E1 (≤8 mg/100 g) [22]. The addition of 4% ALS to the UF resin resulted in a significant decrease in free formaldehyde content by 68.8% compared to the control HDF panel fabricated with 4% UF resin (REF 4). The increased addition of 6% and 8% of ALS in the adhesive formulation resulted in a decrease in formaldehyde content by 73.4% and 78.1%, respectively. The results obtained are in agreement with previous studies on the use of lignosulfonates in wood adhesive applications, which also reported decreased formaldehyde content of the developed composites [65,85,113,114,115].

### 3.4. Thermal Analyses

#### 3.4.1. DSC

**Figure 10:**
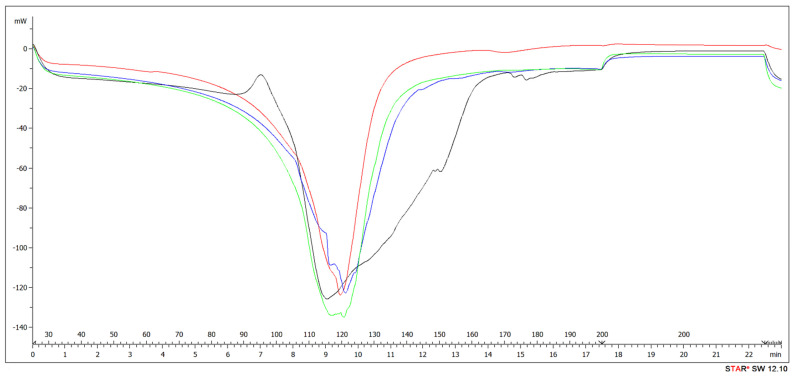
DSC heat of reaction thermograms of adhesive systems (black: UF, red: HDF Type 1, blue: HDF Type 2, green: HDF Type 3).

The DSC method was used to analyze the curing behavior of the adhesives and to explain the effect of ALS addition to UF adhesive mixture (Figure 10, Table 4). The curing temperature is an important parameter because it determines if the adhesive is fully cured during pressing and also in processes like extrusion, such as in carbon fiber production [116,117,118]. The reference sample (UF) had an exothermic curing peak of 110 °C, which can be attributed to the heat released from the polycondensation reaction of primary amino groups of free urea with hydroxymethyl groups [119]. With the addition of ALS, the curing temperature increased regardless of the addition level, which indicates lower reactivity [120]. According to the literature, the curing peak of different lignin types of resins varies between 130 and 150 °C [121,122]. Our results are slightly lower due to UF and due to the hydrophilicity of this lignin type and the slightly retarding effect on curing of adhesive [123,124]. High water absorptivity results in increased viscosity of the adhesive mixture, which reduces the diffusion and mobility of UF resin molecules. Subsequently, their reactive groups decrease with an increase in molecular weight, which results in crosslinking in the curing process [125,126,127,128,129]. The onset of exothermic event was at about 105 °C, which could be indicative of the involvement of carbohydrates being formed.

The reaction enthalpy increased with increasing ALS content up to 8%. Too much ALS partly inhibited the curing reaction of the resin. The lignin part of ALS reacted with free formaldehyde in the adhesive system, leading to its significant decrease as demonstrated in Section 3.3. This result was confirmed in previous research [130,131]. Further decrease in free formaldehyde negatively influenced the reaction enthalpy due to the degree of crosslinking of the resin, which resulted in more active curing of UF [132,133,134].

#### 3.4.2. TGA and DTG

**Figure 11:**
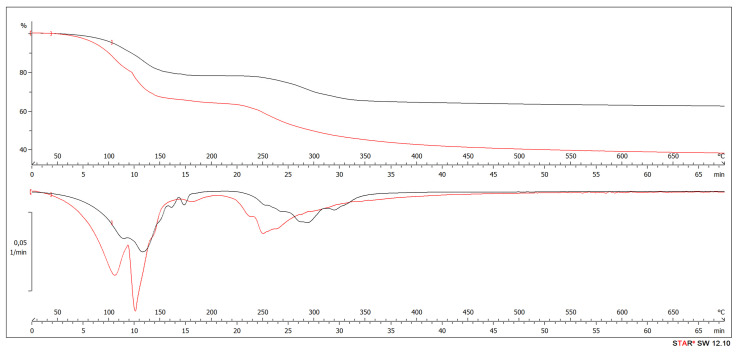
TGA (**top**) and DTG (**bottom**) curves of UF (black) and HDF Type 1 (red) samples.

TGA and DTG analyses were performed to evaluate the effectiveness of ALS addition to UF resin and its influence on thermal stability. The sequence of degradation of adhesive mixtures used in REF 4 and HDF Type 1 panels had two main stages (Figure 11).

The first degradation stage corresponded to mass losses caused by evaporation of water with increasing temperature. Initial mass losses were observed at temperatures below 150 °C, which can be associated with the removal of physically bound moisture from the lignin samples and partial volatilization during polymer chemical modification. The main weight loss occurred due to depolymerization and glass transition of ALS between 170 and 360 °C [135,136] together with formaldehyde and water release due to further reactions between the methylol groups [137,138] and ALS reaction with the free formaldehyde in the system. The visible peak at 175 °C for UF resin might correspond with no absorption of the released formaldehyde by lignin in ALS [139]. The peak temperature at about 250 °C might be associated with degradation of hemicelluloses and, at higher temperatures, with the fragmentation of interunit linkages and release of monomeric phenols [140,141,142].

**Figure 12:**
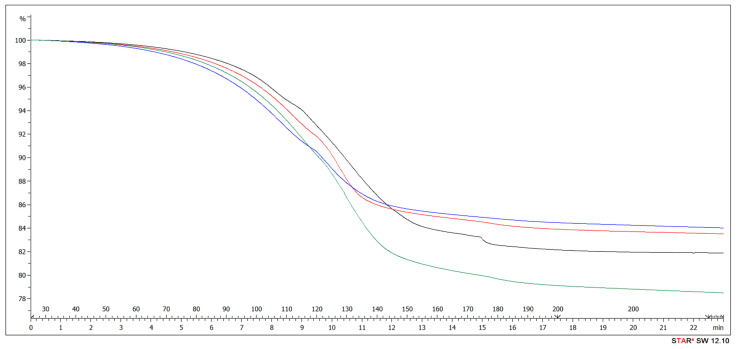
Left part: TGA curves to 200 °C of UF (black: UF, red: HDF Type 1, blue: HDF Type 2, green: HDF Type 3). Right part: isothermal section at the temperature of 200 °C.

Deeper analysis of TGA using the isothermal section was performed to determine the behavior of the resin in case of extended time of pressing at 200 °C. The addition of ALS to UF resin (HDF Type 1 and HDF Type 2 samples provided the best results) significantly reduced the weight loss during the curing of resin at temperatures below 200 °C (Figure 12). No processes other than drying of samples were confirmed.

## 4. Conclusions

Eco-friendly HDF panels with satisfactory physical and mechanical properties and a markedly low free formaldehyde emission can be produced from industrial hardwood fibers bonded with low content (4%) of conventional UF resin and ALS addition levels ranging from 4% to 8% (on the dry wood fibers) under industrial conditions (15 s·mm^−1^). In this research, it was possible to reduce the press factor by 100% by changing the proportions of the input components in the pressing process compared to our previous research [66]. The laboratory-produced HDF panels bonded with 4% and 6% ALS addition fulfilled the strictest standard requirements for HDF panels for use in load-bearing applications. The adhesive formulation composed of 4% UF resin and 4% ALS addition resulted in optimal panel performance. The formaldehyde emission of the panels produced with an adhesive formulation comprising UF resin and ALS additive was very low, varying from 2.0 ± 0.1 mg/100 g to 1.4 ± 0.1 mg/100 g as measured according to Perforator method [88], which allowed for their classification as eco-friendly wood-based panels. The strong formaldehyde scavenging properties of ALS were empirically proven, with a three-fold reduction of free formaldehyde emission being achieved even at the lowest ALS addition level of 4%. The laboratory-fabricated HDF panels with 4% UF resin and ALS content >6% exhibited deteriorated physical and mechanical properties due to the increased moisture content of wood fibers and the short press factor applied (15 s·mm^−1^). The use of lignosulfonates as formaldehyde-free, lignin-based compounds along with conventional UF resins is a promising approach for manufacturing environmentally friendly HDF panels with acceptable properties as sustainable alternatives to traditional wood-based panels. Future studies should be aimed at further optimizing the pressing regimes, modifying the lignosulfonate additives in order to increase their reactivity towards formaldehyde, and systematically investigating the bonding mechanism between the thermosetting resin, lignosulfonate additives, and wood fibers.

## Figures and Tables

**Table 1:** Manufacturing parameters of HDF panels fabricated from industrial hardwood fibers bonded with UF resin and ALS.

HDF Type	Adhesive Type	UF Resin Content, %	Ammonium Lignosulfonate Content, %
REF 4	UF	4	0
HDF Type 1	UF + ALS	4	4
HDF Type 2	UF + ALS	4	6
HDF Type 3	UF + ALS	4	8
REF 6	UF	6	0

**Table 2:** Average dynamic viscosity values (mPa.s) of adhesive formulations used in this work.

UF	UF + 4% ALS	UF + 6% ALS	UF + 8% ALS
23.76 ± 0.52	46.08 ± 0.80	64.40 ± 0.96	66.60 ± 0.98

**Table 3:** Density of HDF panels produced in this work.

Panel Type	REF 4	HDF Type 1	HDF Type 2	HDF Type 3	REF 6
Density, kg·m^−3^	914 ± 17	912 ± 13	910 ± 21	917 ± 18	912 ± 17

**Table 4:** DSC results of adhesive systems used in this work.

	UF	HDF Type 1	HDF Type 2	HDF Type 3
T_onset_ °C	101.44	100.69	103.33	100.5
Tp °C	110.48	115.01	116.18	119.38
∆H J∙g^−1^	815.87	553.89	692.92	826.17

## Data Availability

Not applicable.
